# Two-Stage Hybrid Treatment of Residual Inferior Pancreaticoduodenal Artery Aneurysm Following Treatment of Ruptured Superior Pancreaticoduodenal Artery Aneurysm: A Case Report

**DOI:** 10.3400/avd.cr.25-00018

**Published:** 2025-05-27

**Authors:** Hodaka Wakisaka, Taiki Kakiuchi, Kohei Hachiro, Noriyuki Takashima, Tetsuya Katsumori, Tomoaki Suzuki

**Affiliations:** 1 Department of Cardiovascular Surgery, Saiseikai Shiga Hospital, Ritto, Shiga, Japan; 2 Department of Cardiovascular Surgery, Shiga University of Medical Science, Otsu, Shiga, Japan; 3 Department of Radiology, Saiseikai Shiga Hospital, Ritto, Shiga, Japan

**Keywords:** pancreaticoduodenal artery aneurysm, endovascular treatment, revascularization

## Abstract

Herein, we describe the case of a 72-year-old man who presented with a residual inferior pancreaticoduodenal artery aneurysm following the rupture and treatment of a superior pancreaticoduodenal artery aneurysm. Open surgery for pancreaticoduodenal artery aneurysms requires carefully planned vascular reconstruction to prevent organ ischemia and minimize pressure changes caused by fluctuations in mechanical stress. Additionally, in cases of rupture, factors such as the patient’s condition, presence of hematoma and adhesions, and pressure changes resulting from prior transarterial embolization must be considered. This case report outlines the surgical strategy employed for managing the residual inferior pancreaticoduodenal artery aneurysm.

## Introduction

Visceral artery aneurysms are rare, with pancreaticoduodenal artery aneurysms (PDAAs) accounting for approximately 2% of all cases. PDAA rupture can result in severe clinical consequences, with a high mortality rate of 30%–40%; therefore, appropriate therapeutic intervention is essential.^1,2)^ PDAAs are often associated with stenosis or occlusion of the celiac artery (CA), and aneurysm formation is attributed to the increased pressure from compensatory blood flow through the collateral circulation.^3)^ Treatment guidelines recommend managing PDAAs regardless of aneurysm size. When anatomically feasible, endovascular therapy is the first-line treatment.^4)^ However, even after treatment, if the elevated pressure in the pancreatic arcade caused by collateral circulation persists, there remains a risk of aneurysm recurrence. Furthermore, embolization may lead to ischemia of vital organs. Thus, the treatment approach should be carefully tailored on a case-by-case basis.

There are very few reports on the treatment methods or the optimal timing of intervention for additional aneurysms following the rupture of a visceral artery; these aspects remain controversial. Herein, we present a case of successful 2-stage hybrid treatment for a residual inferior PDAA (IPDAA) following the rupture and treatment of a superior PDAA (SPDAA).

## Case Report

**Figure 1:**
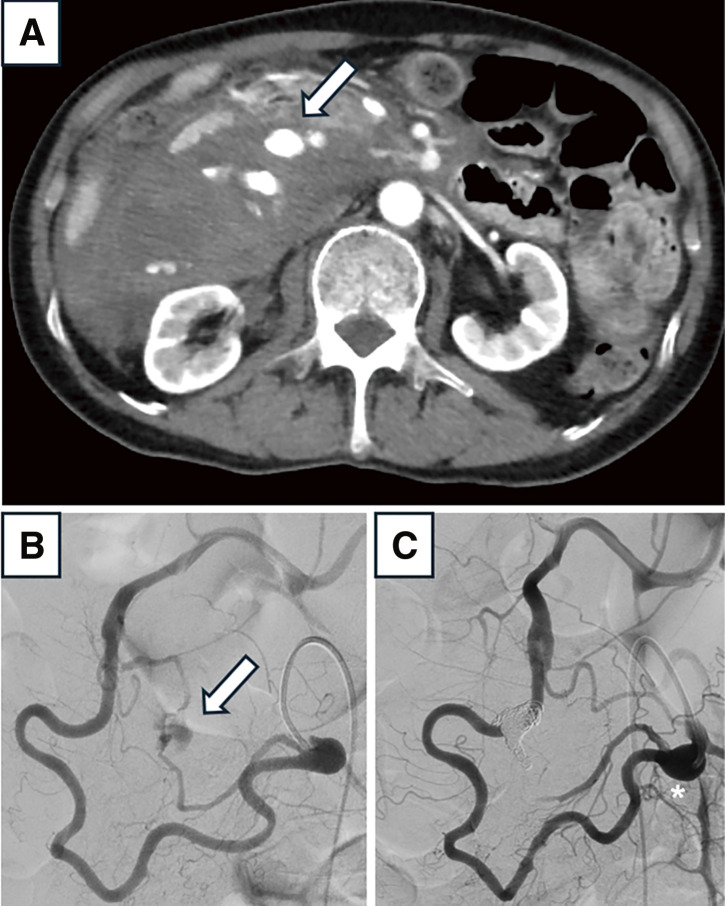
Fig. 1 (**A**) Pre-TAE axial computed tomography angiography showing ruptured SPDAA (arrow) and large intra-abdominal hematoma. (**B**) Angiography via the superior mesenteric artery revealing ruptured SPDAA (arrow). (**C**) Post-TAE angiography showing occlusion of the SPDAA and residual inferior pancreaticoduodenal artery aneurysm (*). SPDAA: superior pancreaticoduodenal artery aneurysm; TAE: transcatheter arterial embolization

A 72-year-old male patient with a medical history of hypertension and panic disorder was urgently brought to our institution due to the sudden onset of upper abdominal pain. His blood pressure was 101/53 mmHg, and heart rate was 114 bpm. Blood tests revealed anemia, with a hemoglobin level of 7.4 g/dL. Computed tomography angiography (CTA) showed a ruptured SPDAA, measuring 15 × 11 mm, accompanied by intra-abdominal bleeding and occlusion at the origin of the CA (**Fig. 1A**). Emergency transcatheter arterial embolization (TAE) was performed to control the rupture, successfully stopping the bleeding and stabilizing the patient (**Figs. 1B** and **1C**). Although an IPDAA, measuring 10 × 10 mm, was also identified, it was the primary source of blood supply to the CA territory. Due to the high risk of ischemia in the upper abdominal organs, coil embolization was not performed. A direct endovascular approach to the CA was attempted; however, blood flow could not be restored. Consequently, open surgical revascularization of the CA region was recommended to ensure an antegrade arterial supply to the upper abdominal organs. This procedure was conducted at another hospital equipped to perform the required surgical intervention.

**Figure 2:**
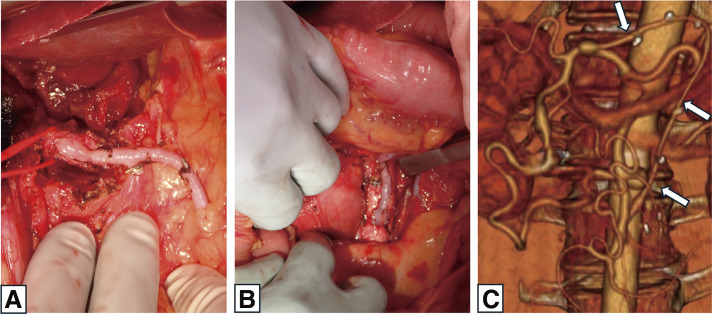
Fig. 2 Distal (**A**) and proximal (**B**) anastomoses of the bypass established from the aorta (inferior to the renal artery) to the common hepatic artery using a great saphenous vein graft. (**C**) Postoperative axial computed tomography angiography confirming graft patency (arrow).

After 3 weeks, a bypass was established from the aorta (inferior to the renal artery) to the common hepatic artery using a great saphenous vein graft. This was performed via a median laparotomy and a transverse approach, with the graft routed through the retroperitoneal space along the dorsal side of the mesentery and the ventral side of the pancreas (**Figs. 2A** and **2B**). The resection of the IPDAA, in a single-stage procedure combined with open surgical bypass, was considered. However, due to adhesions following the rupture and the development of collateral circulation around the aneurysm, it was difficult to identify the aneurysm. Therefore, only the bypass was performed, and a 2-stage hybrid treatment approach was adopted. The postoperative course was uneventful, and CTA confirmed graft patency (**Fig. 2C**). The patient was started on antiplatelet therapy (aspirin) postoperatively to prevent bypass occlusion.

**Figure 3:**
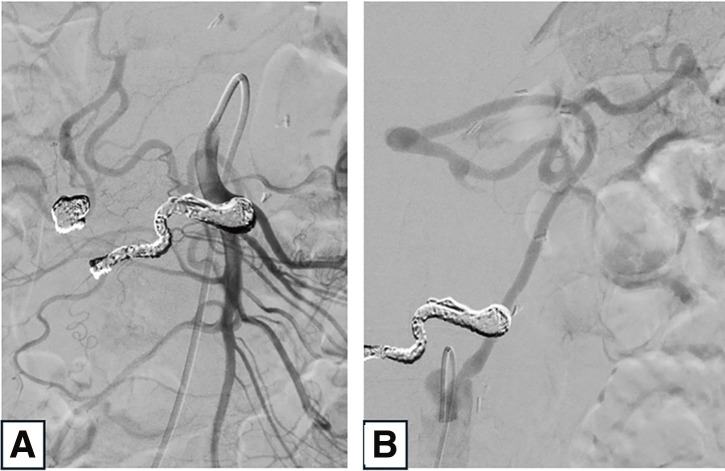
Fig. 3 (**A**) Post-embolization angiography via the superior mesenteric artery showing occlusion of the IPDAA. (**B**) Angiography via the bypass demonstrating graft patency and perfusion to the spleen. IPDAA: inferior pancreaticoduodenal artery aneurysm

Four weeks after the surgery, TAE of the residual IPDAA was performed at our institution. Postoperative imaging confirmed the absence of blood flow within the aneurysm and patency of the bypass, which successfully supplied blood to the upper abdominal organs, particularly the spleen (**Figs. 3A** and **3B**). Ultimately, liver perfusion was maintained through native blood flow from the superior mesenteric artery, but the bypass allowed the second-stage TAE to be performed with reduced risk. At the 1-year follow-up, the bypass remained patent and no new aneurysms had formed.

## Discussion

PDAA is a rare condition, accounting for approximately 2% of all visceral artery aneurysms.^1)^ Compared with other visceral artery aneurysms, PDAAs have a higher risk of rupture; therefore, treatment is recommended regardless of aneurysm size. Endovascular treatment is the primary approach for managing PDAAs; however, in anatomically challenging cases, open surgery may be required.^4)^ Notably, approximately 70% of PDAAs are associated with CA occlusion or stenosis, and the resulting increase in collateral circulation from the superior mesenteric artery contributes to aneurysm formation.^3,5)^ Miyahara et al. reported that due to CA stenosis or occlusion, blood flow to the pancreatic arcade is approximately 3 times higher than normal.^6)^

Furthermore, when considering embolization, it is crucial to evaluate organ perfusion on a case-by-case basis and ensure that adequate revascularization is achieved.

In this case, after performing TAE for a ruptured SPDAA, open surgery was conducted for an unruptured IPDAA. This approach was taken because TAE of the IPDAA could have led to ischemia in the upper abdominal region due to CA occlusion. Therefore, a bypass was created in advance to maintain adequate perfusion. There are very few reports on the early open surgical intervention for residual aneurysms following the rupture of visceral artery aneurysms. To the best of our knowledge, no studies specifically address PDAAs in this context. Murase et al. reported surgical intervention for splenic artery aneurysms after the rupture of visceral artery aneurysms.^7)^ However, in cases of PDAA, concerns such as postoperative adhesions and the risk of pancreatic fistula often lead to a preference for delayed TAE.

Several reports have shown that changes in arcade blood flow after embolization can lead to the enlargement, rupture, or formation of new aneurysms. For instance, in the study by Hasegawa et al., a new aneurysm ruptured 27h after TAE, indicating that aneurysms can form at a very early stage.^8)^ Therefore, early intervention is highly recommended whenever possible. In contrast, in cases where the patient’s overall condition has deteriorated due to rupture, or if open surgery is difficult to perform in the early stages due to abdominal hematoma or adhesions, intervention may be challenging. Thus, the optimal timing for open surgical intervention remains controversial. In the present case, surgery was performed 3 weeks after the rupture, once the patient’s condition had improved. By that time, the residual aneurysm remained stable, even though the hematoma was still present in the abdominal cavity. We planned to perform reconstruction using a saphenous vein graft to create a bypass from the abdominal aorta to the common hepatic artery. However, because the abdominal aorta might be difficult to access, we also planned to use the splenic artery or the middle colic artery as alternative in situ bypass vessels if required.^9)^ Additionally, if possible, we intended to complete the treatment by performing aneurysm resection after establishing the bypass. However, due to adhesions and the development of collateral circulation around the aneurysm, which posed a risk of bleeding, we decided to carry out the intervention in 2 stages, with the second stage planned for a later date. In cases of surgical intervention after rupture, it is important to anticipate unexpected situations and consider various options.

In the treatment of visceral artery aneurysms associated with median arcuate ligament (MAL) syndrome, the need to perform additional MAL resection remains a topic of debate. If aneurysms occur due to the aforementioned mechanism—namely, CA stenosis—the risk of aneurysm recurrence persists unless the underlying cause is addressed. There are only a few reports on aneurysm recurrence in the long term after TAE; nevertheless, the majority of these reports indicate no recurrence.^10)^ In the present case, MAL resection was not performed to keep the procedure as minimally invasive as possible. At the 1-year postoperative stage, no aneurysm recurrence was observed; however, continuous follow-up remains necessary.

Maeda et al. showed that revascularization can reduce pressure in the arcade—a collateral blood channel—which may lead to mass reduction.^10)^ If this is the case, TAE should not be performed as the initial treatment following revascularization; rather, the patient should be closely monitored through follow-up. During this period, however, due to the risk of rupture and bypass occlusion caused by abundant blood flow on the distal side of the bypass vessel—which was experiencing to-and-fro blood flow—he residual aneurysm was managed with early TAE.

## Conclusion

A 2-stage treatment procedure involving open surgery and TAE was performed for a residual IPDAA that developed after the rupture and treatment of an SPDAA. Vascular reconstruction should be performed with careful consideration to prevent organ ischemia caused by reduced blood flow in the CA region and to manage pressure changes resulting from fluctuations in mechanical stress due to decreased regional perfusion. Furthermore, when addressing a residual aneurysm after the treatment of a ruptured visceral artery aneurysm, it is essential to consider various factors—including the patient’s condition, the presence of hematoma and adhesions, and pressure changes caused by prior TAE—in determining the appropriate timing and type of surgical intervention.

## Declarations

### Informed consent

Informed consent was obtained from the participant for the publication of this case report.

### Patient consent

We have obtained patient consent for this study.

### Acknowledgments

None.

### Disclosure statement

All authors have no conflicts of interest to declare.

### Author contributions

Study conception: HW

Writing: HW

Critical review and revision: all authors

Final approval of the article: all authors

Accountability for all aspects of the work: all authors.
